# A Correlation Between *Pericarpium Citri Reticulatae* Volatile Components and the Change of the Coexisting Microbial Population Structure Caused by Environmental Factors During Aging

**DOI:** 10.3389/fmicb.2022.930845

**Published:** 2022-07-22

**Authors:** Fangqing Yang, Liying He, Mengyuan Shen, Fu Wang, Hongping Chen, Youping Liu

**Affiliations:** College of Pharmacy, Chengdu University of Traditional Chinese Medicine, Chengdu, China

**Keywords:** *Pericarpium Citri Reticulatae*, aging, environmental factors, population structure of coexisting microorganisms, volatile components

## Abstract

*Pericarpium Citri Reticulatae* (PCR) is a natural citrus by-product with beneficial health and nutritive properties that is used widely in food and is an ingredient in traditional Chinese medicine. PCR improves gradually with aging. However, the present research has not yet revealed the reasons for this. Some data prove the important role of microorganisms in the quality of tobacco and fermented tea with the time of the aging of these foods. Our studies further proved that the coexisting *Aspergillus niger* plays an important role in the change of flavonoids and volatile oil in PCR during this process. Therefore, we put forward that longer storage is better for PCR and is highly correlated with the change of the coexisting microbial population structure caused by environmental factors. Samples of PCR aged in Beijing, Sichuan, Guangdong, and Yunnan were collected at different time points. Using GC/MS and high throughput 16S rDNA and ITS sequencing techniques, massive changes in volatile profile and microbial communities were observed during aging. Spearman correlation analysis indicated that *Exobasidium, Xeromyces, Pseudocercospora, Russula, Aspergillus, Herbaspirillum, Sphingomonas*, and *Streptococcus*, which are the dominant microbial genera in Sichuan and Guangdong showed strong connections with volatile components of chemical markers. It was preliminarily verified that the changes of volatile components for PCR are highly correlated with the change of the coexisting microbial population structure caused by environmental factors, providing a new idea for the research on the aging mechanism of PCR and key influencing factors of aging quality.

## Introduction

PCR is the dried peel of the citrus fruit, *Citrus reticulata Blanco*, and its cultivated varieties. These peels are prepared typically from freshly picked fruits that are permitted to dry in the sun or at low temperatures. PCR is a staple in foods and drugs due to its effectiveness in regulating the qi (energy), normalizing the functions of the spleen and stomach, and resolving the phlegm. To quote Tao Hongjing (Tao, 1955) “The longer the orange peel is stored, the better the quality will be.” At present, there is no research on the process of improving the PCR quality with longer storage. The most common literature data deal with the study of variations in its chemical components and the strength of pharmacological effects in the aging process (Wang et al., 2017; Yang et al., 2021a,b), and there is little research on its aging mechanism.

Microorganisms play an important role in the aging quality formation of tobacco and fermented tea. The main cause of the formation of flavor substances is the change in the coexisting microbial population structure and metabolic enzyme, storage temperature and humidity, time, and variations of components (Yang et al., 2006; Zhao et al., 2009; Liu et al., 2010; Tian et al., 2013; Li et al., 2014; Wang et al., 2015a,b). Our data highlighted that the coexisting *Aspergillus niger* played an important role in the change of flavonoids and volatile oil in PCR (Wang et al., 2015; Zhang et al., 2017a,b). Other literature data reported that *Bacillus, Lactococcus*, and *Pseudomonas* can accelerate the biotransformation of volatile oil in PCR (Wang et al., 2015; Chen, 2017; He, 2019). *Bacillus*, S*tenotrophomonas, Acidisoma, Sphingomonas, Enterobacter, Neochlamydia*, and *Methylobacterium* were associated with a variety of volatile oil components in PCR (He, 2019). These studies confirmed that the microorganisms on the surface of PCR could cause the quality change of PCR. However, the relationship between the changes in microbial population structure and environmental factors during the aging process of PCR was not studied. A correlation of *Pericarpium Citri Reticulatae* (PCR) between volatile components and the change of the coexisting microbial population structure caused by environmental factors is essential to understanding the underlying processes of the improvement of PCR with the time of its storage and the microbiota that drives this.

High-throughput sequencing technology is characterized by high sequencing depth, convenient identification of low-abundance community species, and low cost (Caporaso et al., 2012; Degnan and Ochman, 2012). It has been widely used in microbial diversity analysis of wine (Lu et al., 2020; Deng et al., 2021), sufu (Huang et al., 2018a), and food fermentation (Lv et al., 2019; Yi et al., 2019). It provides a reliable and effective method for diversity analysis of microbial community structure in environmental samples (Tringe et al., 2005).

In this study, *Citrus reticulata* “Chachi” samples were selected under the conditions of the fixed area, variety, harvest time, and processing method. Samples of *Citrus reticulata* “Chachi” aged in Beijing, Sichuan, Guangdong, and Yunnan were collected at different time points. High-throughput sequencing of fungal ITS 1 (Internal Transcribed spacer 1) and bacterial 16S rDNA V4 was performed to compare the microbial community structure. The GC/MS method was applied to the analysis of main differential components. Furthermore, we applied stoichiometry and other analysis methods to analyze the correlation of microbial-volatile components. The study provides a new idea for the research on the aging mechanism of *Citrus reticulata* “Chachi” and lays a foundation for recommendations for PCR storage, maintenance, and quality evaluation.

## Materials and Methods

### Sample Collection

To ensure that the collected PCR samples were representative and uniform, all specimens were collected in early January 2020 from the fresh oranges stored for peeling in Xinmadan Village, Sanjiang Town, Xinhui District, Jiangmen City, and Guangdong Province, China. After peeling, the product was processed and dried traditionally. Specifically, the orange peels were placed under different environmental conditions like wind, sun, and natural loss of water. The drying process included the following steps: soft texture after turning the peel, turning the peel then and finishing the process by drying it. The samples were identified as *Citrus reticulata* “Chachi” by Professor Yan Zhuyun from the Department of Traditional Chinese Medicine, College of Pharmacy, Chengdu University of Traditional Chinese Medicine. A linen bag containing the orange peels was placed in the cool, dry, and ventilated warehouse within the 302 Hospital of people's liberation army, No. 100, Middle West Fourth Ring Road, Fengtai District, Beijing City; the pharmacy building of the Chengdu University of Traditional Chinese Medicine, Wenjiang District, Chengdu City, Sichuan Province; No. 208, Didong Road, Pengjiang District, Jiangmen City, Guangdong Province; the Yunnan University of Traditional Chinese Medicine, Chenggong District, Kunming City, Yunnan Province. This allowed the product to age naturally. The same number of samples were removed from the linen bag every 3 months of storage in Sichuan and Guangdong, and every 6 months in Beijing and Yunnan. Samples were stored at −20°C before analysis of volatile compounds and were deposited at −80°C before DNA extraction. The details of the samples are shown in Table 1.

**Table 1:** Information of *Citrus reticulata* “Chachi” samples.

**Sample number**	**Varieties**	**Aging areas**	**Collecting times**	**Aging time**
GD0	*Citrus reticulata* “Chachi”	Guangdong, China	January 22, 2020	Fresh peel
SC1	*Citrus reticulata* “Chachi”	Sichuan, China	June 4, 2020	4 months
GD1	*Citrus reticulata* “Chachi”	Guangdong, China	June 4, 2020	4 months
BJ2	*Citrus reticulata* “Chachi”	Beijing, China	July 28, 2020	6 months
SC2	*Citrus reticulata* “Chachi”	Sichuan, China	July 28, 2020	6 months
GD2	*Citrus reticulata* “Chachi”	Guangdong, China	July 28, 2020	6 months
YN2	*Citrus reticulata* “Chachi”	Yunnan, China	July 28, 2020	6 months
SC3	*Citrus reticulata* “Chachi”	Sichuan, China	November 3, 2020	9 months
GD3	*Citrus reticulata* “Chachi”	Guangdong, China	November 3, 2020	9 months
BJ4	*Citrus reticulata* “Chachi”	Beijing, China	February 1, 2021	12 months
SC4	*Citrus reticulata* “Chachi”	Sichuan, China	February 1, 2021	12 months
GD4	*Citrus reticulata* “Chachi”	Guangdong, China	February 1, 2021	12 months
YN4	*Citrus reticulata* “Chachi”	Yunnan, China	February 1, 2021	12 months

**Figure 1:**
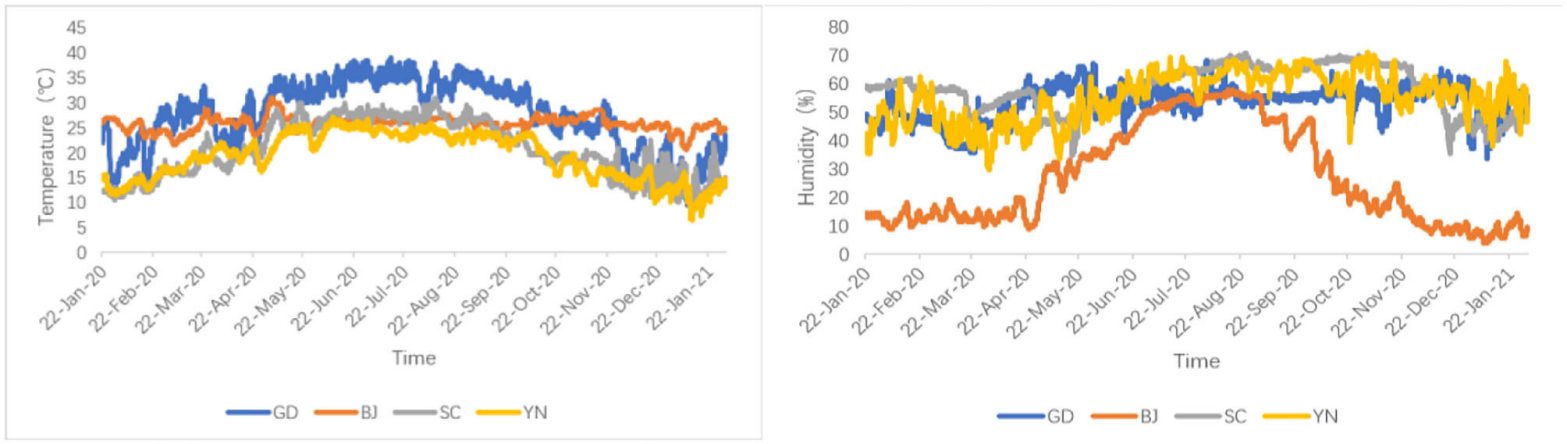
Temperature and humidity curve of the aging process in different areas.

The environmental factors (temperature and humidity) in the aging process of the dried orange peel were recorded by a DS 1923 button (Wdsen Electronic Technology Co., Ltd) type temperature-humidity recorder. All these parameters are shown in Figure 1.

### High Throughput Sequencing Analysis

The DNA was extracted by referring to our previous methods (Zhang et al., 2017a,b; Yang et al., 2021a,b), The diluted genomic DNA was used as a template for PCR amplification with barcode-containing specific primers, the Phusion^®^ High-Fidelity PCR Master Mix with GC Buffer, and a high-efficiency high-fidelity enzyme. The V4 regions of the bacterial 16S rRNA gene and the internal transcribed spacer (ITS1) regions of fungal rRNA genes were amplified using the primers 515F (5′-GTGCCAGCMGCCGCGGTAA-3′)/806R (5′-GGACTACHVGGGTWTCTAAT-3′) and ITS1-1F-F (5′-CTTGGTCATTTAGAGGAAGTAA-3′) /ITS1-1F-R (5′-GCTGCGTTCTTCATCGATGC-3′) with barcodes, respectively (Wang et al., 2020). The PCR reactions (30 μl) included the Phusion Master Mix (2x; 15 μl), positive and negative primers (2 μmol/L; each at 1.5 μl), genomic DNA (gDNA; 1 mg/L, 10 μl), and ddH2O (2 μl). The bacterial PCR cycling conditions were 98°C for 1 min, followed by 30 cycles of 98°C for 10 s, 50°C for the 30 s, 72°C for 30 s, and finally, 72°C for 5 min. The fungal PCR cycling conditions were 98°C for 30 s, followed by 35 cycles of 98°C for 10 s, 56°C for the 30 s, 72°C for 15 s, and finally, 72°C for 7 min. The PCR product was detected by 2% agarose gel electrophoresis. The amplified product was visualized by a gel recovery kit. A library was constructed using a TruSeq DNA PCR-Free Library Preparation Kit. The library was evaluated by Qubit and Q-PCR and subjected to sequencing using NovaSeq 6000. The data presented in the study are deposit ed in the NCBI repository, accession number are PRJNA854783 and PRJNA854691.

### Steam Distillation for Volatile Oil

About 50.00 g PCR of coarse powder was put into a 1,000 ml distillation flask. About ten times that amount of water was added. Samples were soaked for 1 h. Volatile oil distillation apparatus was set according to Chinese Pharmacopeia. The oil was distilled, obtained from the condenser, and dried over anhydrous sodium sulfate at 4°C for 24 h; 50 μl of volatile oil from each sample was taken and put into a 1 ml volumetric flask with the solution to the scale line with n-hexane. The samples were then filtered through a 0.22 μm filter and were analyzed.

### Volatile Profiles by GC-MS

The GC-MS technique was used in this study to identify the volatile. The column was an HP-5MS quartz capillary column (0.25 mm×30 m), 0.25 μm. The oven temperature was programmed as follows: the initial column temperature was 60°C, with 2°C /min rise to 82°C, then 20°C /min rise to 144°C (to keep 3 min), 8°C /min rise to 260°C (to keep 10 min). The carrier gas He was in the constant current mode, the flow rate was 1.0 ml/min, the split ratio was 10:1, and the autosampler was 1.0 μl. The ion source was electrospray (EI), the ionization energy injector temperature was 270°C, the transmission line temperature was 240°C, and the solvent delay was 9 min.

### Data Analysis

The barcode sequences and PCR amplification primer sequences were removed from each data file. Reads from each sample were spliced using the FLASH v1.2.7 software. The spliced sequences obtained were the raw tags. These files were filtered to obtain the clean tags (Bokulich et al., 2013). The quality of the tags was evaluated with the QIIME v1.9.1 software (Caporaso et al., 2010). The processed sequence tags were compared with those present in the unit database using the UCHIME algorithm to detect chimeric sequences. The chimeric sequences were removed (Bokulich et al., 2013) to obtain the final list of effective tags. The operational taxonomic unit (OTU) clustering and species annotation was performed by UPARSE v7.0.1001 (Caporaso et al., 2010) software to generate clusters from all the effective tags from the samples. The sequences were clustered into OTUs with 97% identity. ITS sequencing data analyses were done following annotation analysis of representative OTU sequences by the BLAST method and the Unit database in QIIME software. 16S sequencing data were analyzed by species annotation analysis of representative OTU sequences performed by the MOTHUR method and SSU rRNA database (Edgar, 2013) in the QIIME software. The community composition of each sample was determined at each classification level.

Alpha and beta diversity was assessed by the QIIME software, used to calculate the observed species by the Shannon and abundance-based coverage estimation (ACE) diversity plot and the Chao index. The goods coverage indices and the unweighted pair-group method were assessed by the arithmetic means (UPGMA) and were used to construct the UPGMA sample clustering tree. The R software (V2.15.3) was used to draw the petal diagram and non-metric multidimensional scale (NMDS). Volatile components were identified by the NIST MS Search Program (version 2.0g). The similarity of volatile compounds was detected in over 80% of the standard spectra library. The relative percentage content of each component was calculated by the area normalization method. SIMCA-P 14.0 (Umetrics, Umeå, Sweden) was used to perform principal component analysis (PCA) together with the orthogonal partial least squares discriminant analysis (OPLS-DA), and the differential chemical markers were estimated by using variable importance in projection (VIP) values. Spearman's correlation was used to conduct analyses of volatiles and microorganisms. The experiment was carried out in three technical replicates to ensure the reliability of the data.

## Results

### Abundance and Diversity of Microbial Communities in Different Samples

**Figure 2:**
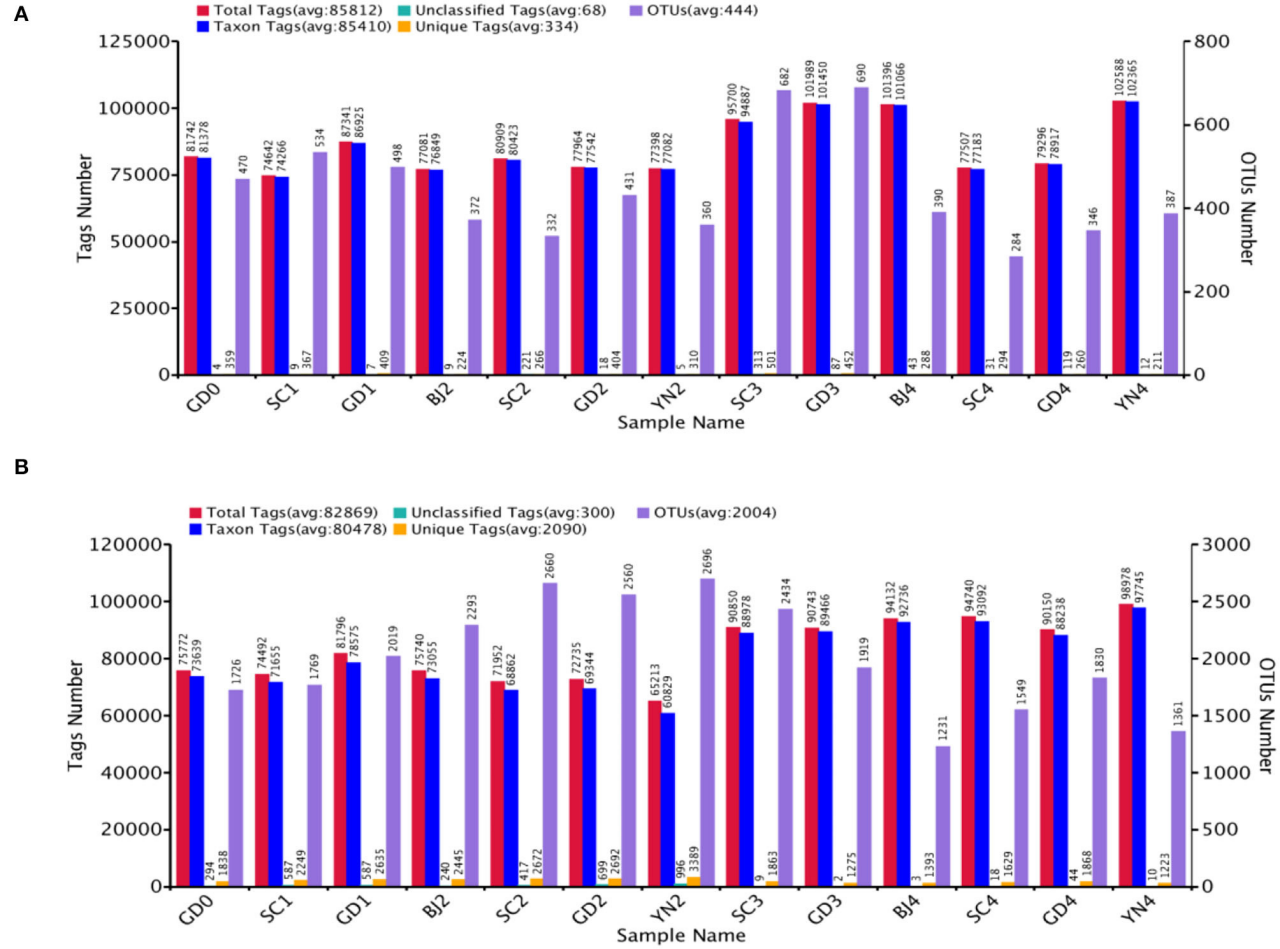
**(A)** The number of tags and OTUs of fungi in *Citrus reticulata* “Chachi” samples. **(B)** The number of tags and OTUs of bacteria in *Citrus reticulata* “Chachi” samples. Total Tags: the total number of stitching sequences after filtering, Taxon Tags: the number of tags that build OTUs and provide information about classification, Unclassified Tags: the number of tags that build OTUs but do not provide any information about classification, Unique Tags: the number of tags with a frequency of 1 which cannot be clustered into OTUs, OTUs: the final number of OTUs.

Total fungal tags 1 115 554 with an average of 85 812 tags per sample, and 1 077 293 bacterial tags with an average of 82 869 tags per sample using the tags quality control process of the Qiime (v1.7.0) software were performed. Sequences were grouped into 5 776 OTUs for fungi and 26 047 OTUs for bacteria (both at 97% similarity level), as shown in Figure 2.

**Figure 3:**
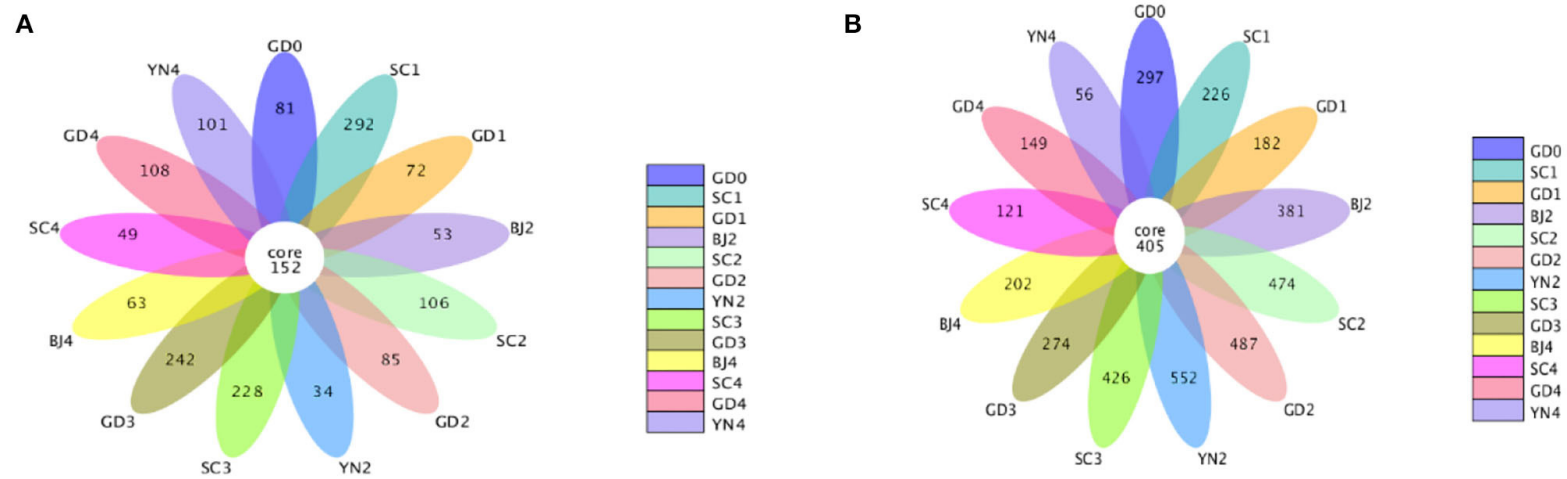
**(A)** Petal diagram of the fungal community present in *Citrus reticulata* “Chachi” samples. **(B)** Petal plot of the bacterial community present in *Citrus reticulata* “Chachi” samples.

We applied indices of community richness, including Shannon, Simpson, Chao, and ACE indexes. Shannon and Simpson indexes were used to estimate the diversity of the microbial community, while the Chao1 and ACE estimators were used to estimate the microbial richness of samples (Zhang et al., 2017a,b). The Shannon and Simpson indexes had no statistical differences (*p* > 0.05), while the Ace and Chao ones had statistical differences (*p* < 0.05) with larger values indicating greater abundance. The combined data revealed an increase in the abundance and number of distinct fungal species on the surface of the PCR in its samples stored for 9 months of aging, where the temperature and humidity underwent a gradual increase. The combined data revealed an increase in the abundance and number of distinct bacterial species on the surface of the PCR in the 6 and 9 months of aging, with the temperature, and humidity undergoing a gradual increase (Table 2). The warm and moist climate facilitated fungal growth on the PCR surface. Bacterial OTUs were estimated to be higher than that fungal OTUs as shown in the Petal diagram in Figures 3A,B. The petal diagram for fungi further showed that 152 OTUs from the core microflora were detected in all samples, among which the number of unique OTUs was more abundant in the samples of 9 months of aging. As far as bacteria are concerned, the petal diagram showed that 405 OTUs belonged to the core microflora and they were detected in all samples, among which the number of unique OUTs was bigger when the samples were after 6 and 9 months of aging.

**Table 2:** Diversity index of the microbial community in *Citrus reticulata* “Chachi” samples.

**Sample Name**	**Observed_species**		**Shannon**		**Simpson**		**Chao1**		**ACE**		**Goods_coverage**	
	**1**	**2**	**1**	**2**	**1**	**2**	**1**	**2**	**1**	**2**	**1**	**2**
GD0	412	1 458	4.449	3.831	0.916	0.728	478.683	1 685.961	498.212	1 779.652	0.998	0.993
SC1	485	1 483	4.298	4.346	0.865	0.787	526.677	1 761.144	546.578	1 866.057	0.998	0.992
GD1	435	1 665	3.412	4.655	0.667	0.812	559.726	2 130.028	553.595	2 210.31	0.998	0.99
BJ2	320	1 972	3.489	5.098	0.785	0.832	364.381	2 274.621	382.842	2 407.736	0.999	0.99
SC2	295	2 313	4.673	5.634	0.935	0.873	369.643	2 651.456	359.187	2 763.752	0.999	0.989
GD2	390	2 207	4.801	4.935	0.93	0.829	430.686	2 500.603	443.076	2 628.825	0.999	0.99
YN2	310	2 502	4.041	5.146	0.885	0.795	372.258	3 237.379	384.949	3 116.296	0.998	0.987
SC3	594	2 078	4.88	5.407	0.916	0.847	770.539	2 593.181	788.37	2 687.617	0.996	0.988
GD3	603	1 605	4.357	4.183	0.846	0.757	794.865	2 177.283	816.153	2 190.938	0.996	0.99
BJ4	339	1 065	4.468	4.188	0.911	0.752	418.684	1 354.367	421.046	1 355.464	0.998	0.994
SC4	255	1 335	4.16	5.25	0.89	0.873	274.544	1 660.767	278.721	1 675.142	0.999	0.993
GD4	326	1 569	4.786	5.474	0.918	0.869	373.058	1 943.018	362.902	1 985.642	0.999	0.992
YN4	341	1 131	4.176	3.69	0.875	0.741	406.008	1 509.294	413.747	1 535.635	0.999	0.993

*1: fungi, 2: bacteria*.

### Comparison of Fungal Communities in Different Samples

**Figure 4:**
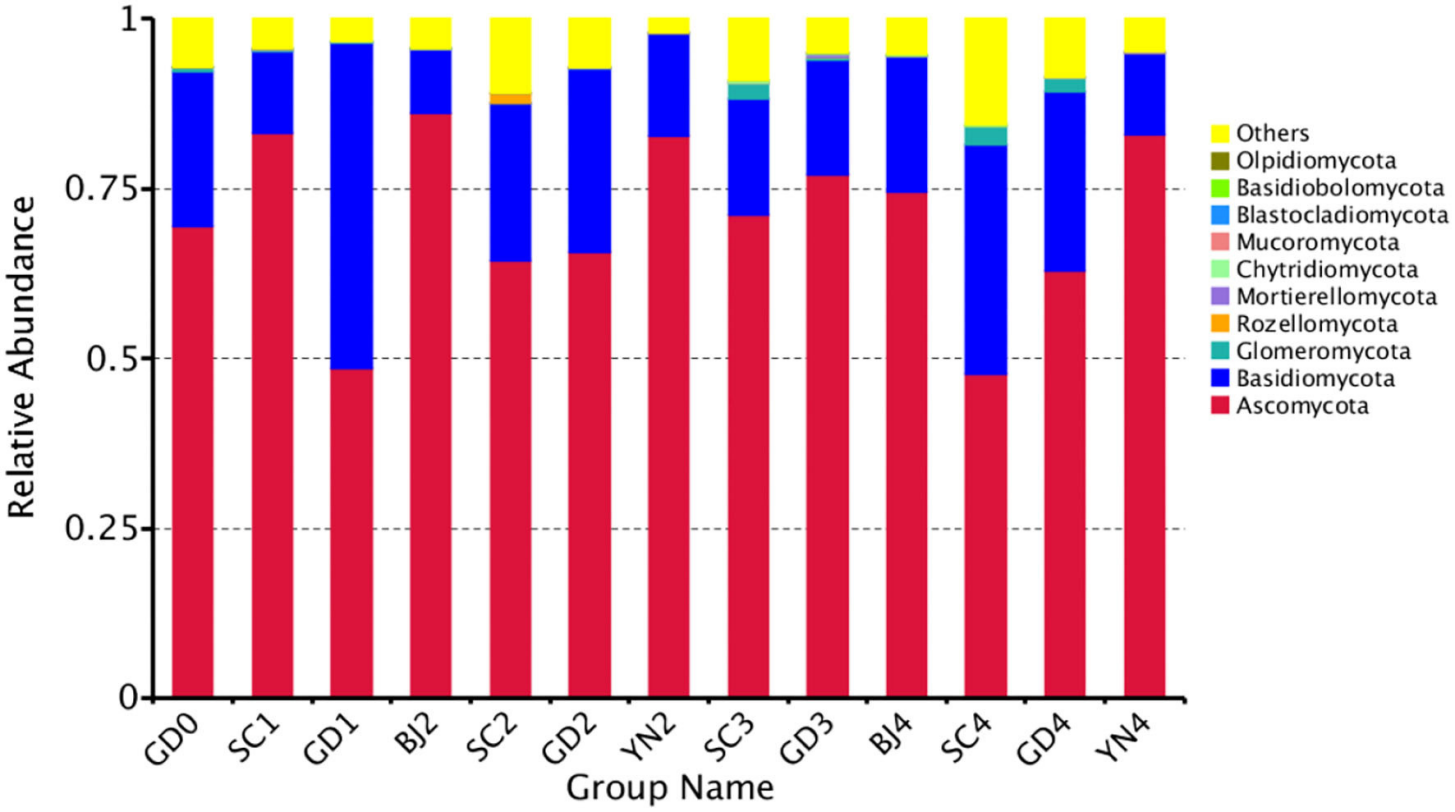
Relative abundance of fungi at phylum levels. Distribution of 10 most abundant fungal phylum present in *Citrus reticulata* “Chachi” samples. “Others” in the last column refers to the sum of less abundant fungal phylum. Each number represents average of three replicates.

The composition of the fungal communities identified on the PCR surface at each sampling point was analyzed and identified. It was found that the fungal community composition on the surface of PCR was similar, but the abundance of the fungal communities changed significantly during aging. At the phylum level, these fungi were distributed mainly among the *Ascomycota* (70.5%) and *Basidiomycota* (21.85%), with smaller populations of *Glomeromycota, Rozellomycota, Mortierellomycota, Chytridiomycota, Mucoromycota, Blastocladiomycota, Basidiobolomycota*, and *Olpidiomycota*, among others (Figure 4).

**Figure 5:**
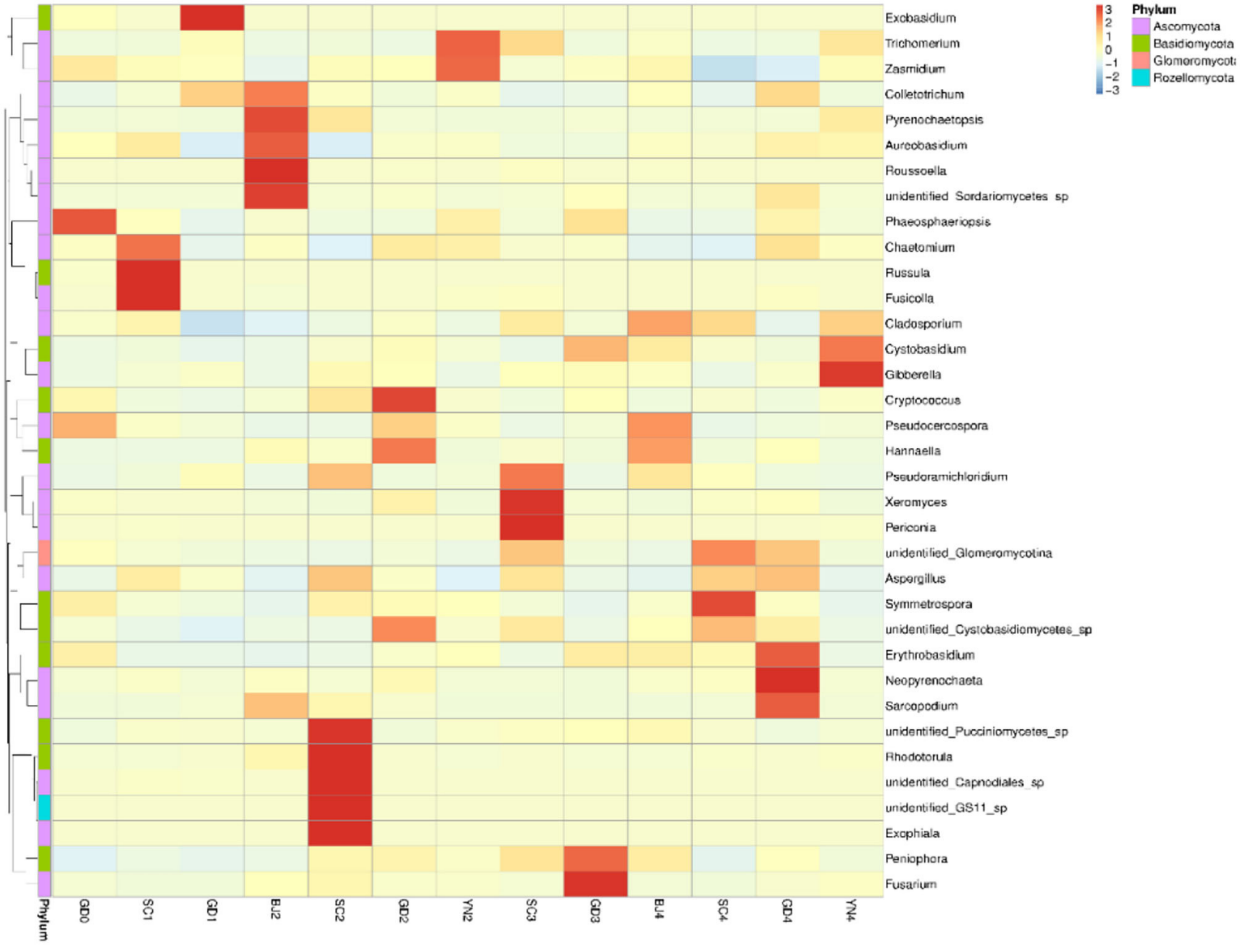
Heatmap depicting relative species abundance. Plotted by sample name on the X-axis and the Y-axis represents the genus. The cluster tree on the left side of the graph is a species cluster tree.

According to the species annotation and abundance information at the genus level (Figure 5), together with the abundance information associated with each sample, the top 35 identified genera were selected and clustered in a heat map. The species of Z > 1 in the heat map were defined as dominant fungal genera and were plotted as a table. According to the results in Table 3 and Figure 5, all the known fungi of the genera, *Phaeosphaeriopsis* and *Pseudocercospora* were predominant in the fresh citrus peels, while the genus *Fusicolla, Russula*, and *Chaetomium* were mainly distributed in the SC1. The fungi of the genera *Colletotrichum* and *Exobasidium* were predominant in GD1. *Colletotrichum* and *Sarcopodium* appeared more abundant when the samples were stored for BJ2. *Rhodotorula* and *Aspergillus* appeared more abundant when samples were stored for SC2. *Cryptococcus* and *Hannaella* were predominant in GD2. *Trichomerium* and *Zasmidium* were principal in YN2. *Periconia* and *Xeromyces* were found in SC3, while *Fusarium, Peniophora*, and *Cystobasidium* were more abundant when samples were stored for GD3. *Pseudocercospora, Hannaella* and *Cladosporium* were found in BJ4. *Aspergillus* and *Cladosporium* were predominant in SC4. *Neopyrenochaeta* and *Aspergillus* were predominant in GD4, while *Gibberella, Cystobasidium*, and *Cladosporium* gathered more when samples were stored for YN4. Noteworthy, our findings highlighted that the coexisting *Aspergillus niger* played an important role in the change of flavonoids and volatile oil in PCR (Wang et al., 2015; Zhang et al., 2017a,b). *Aspergillus* is also proven to be the dominant fungal genera on the surface of PCR aged in Sichuan and Guangdong areas.

**Table 3:** Distribution of the dominant fungal genera in all samples.

**Sample name**	**The dominant fungal genera**
GD0	*Phaeosphaeriopsis, Pseudocercospora*
SC1	*Fusicolla, Russula, Chaetomium*
GD1	*Colletotrichum, Exobasidium*
BJ2	*Colletotrichum, Sarcopodium, Aureobasidium, Pyrenochaetopsis, Roussoella*
SC2	*Rhodotorula, Exophiala, Aspergillus, Pseudoramichloridium*
GD2	*Cryptococcus, Hannaella, Pseudocercospora*
YN2	*Trichomerium, Zasmidium*
SC3	*Periconia, Xeromyces, Pseudoramichloridium, Trichomerium*
GD3	*Fusarium, Peniophora, Cystobasidium*
BJ4	*Pseudocercospora, Hannaella, Cladosporium*
SC4	*Symmetrospora, Aspergillus, Cladosporium*
GD4	*Neopyrenochaeta, Erythrobasidium, Sarcopodium, Aspergillus, Colletotrichum*
YN4	*Gibberella, Cystobasidium, Cladosporium*

**Figure 6:**
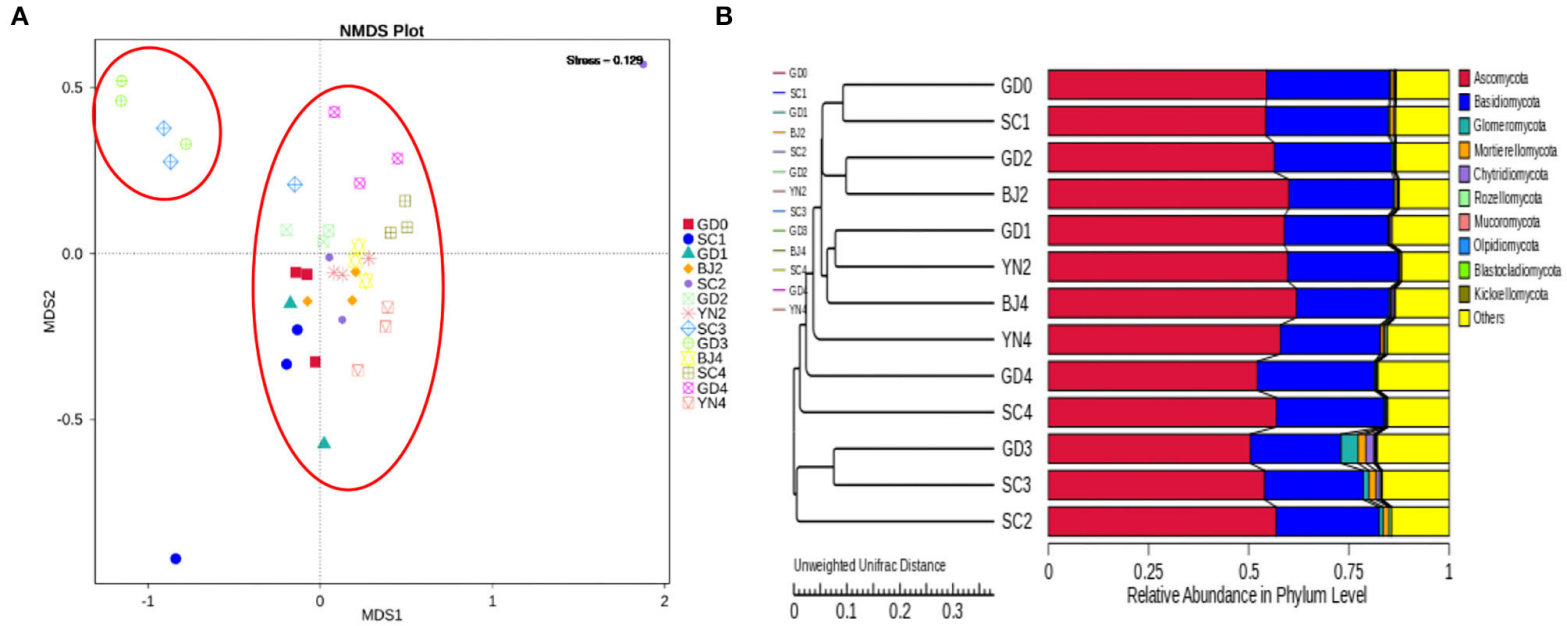
**(A)** NMDS analysis of fungal community composition. Each point represents a sample, plotted by the second principal component on the Y-axis and the first principal component on the X-axis, which was colored by group. **(B)** UPGMA clustering tree based on weighted UniFrac distance. UPGMA clustering tree (at left). The relative phylum-level abundance map (at right).

An NMDS analysis reflected the identification of fungi contained in each PCR specimen in a multidimensional space in the form of dots. The NMDS analyses concerning the OTU levels are shown in Figure 6A. We observed that when the community composition of the samples was more similar, they fell closer to one another in the NMDS diagram. As shown in Figure 6A, SC3 and GD3 were distant from those representing the other samples. The distances between the remaining samples were relatively small. To evaluate the similarities and differences between these samples, we carried out cluster analysis and constructed a cluster tree. Weighted and unweighted UniFrac distance matrices were used as the basis for UPGMA clustering analysis. The clustering results were integrated with the relative abundance data, as shown in Figure 6B. According to the UPGMA clustering tree results, the composition of the fungal species was similar during the aging process and, as such, could be evaluated as a single group.

### Comparison of Bacterial Communities in Different Samples

**Figure 7:**
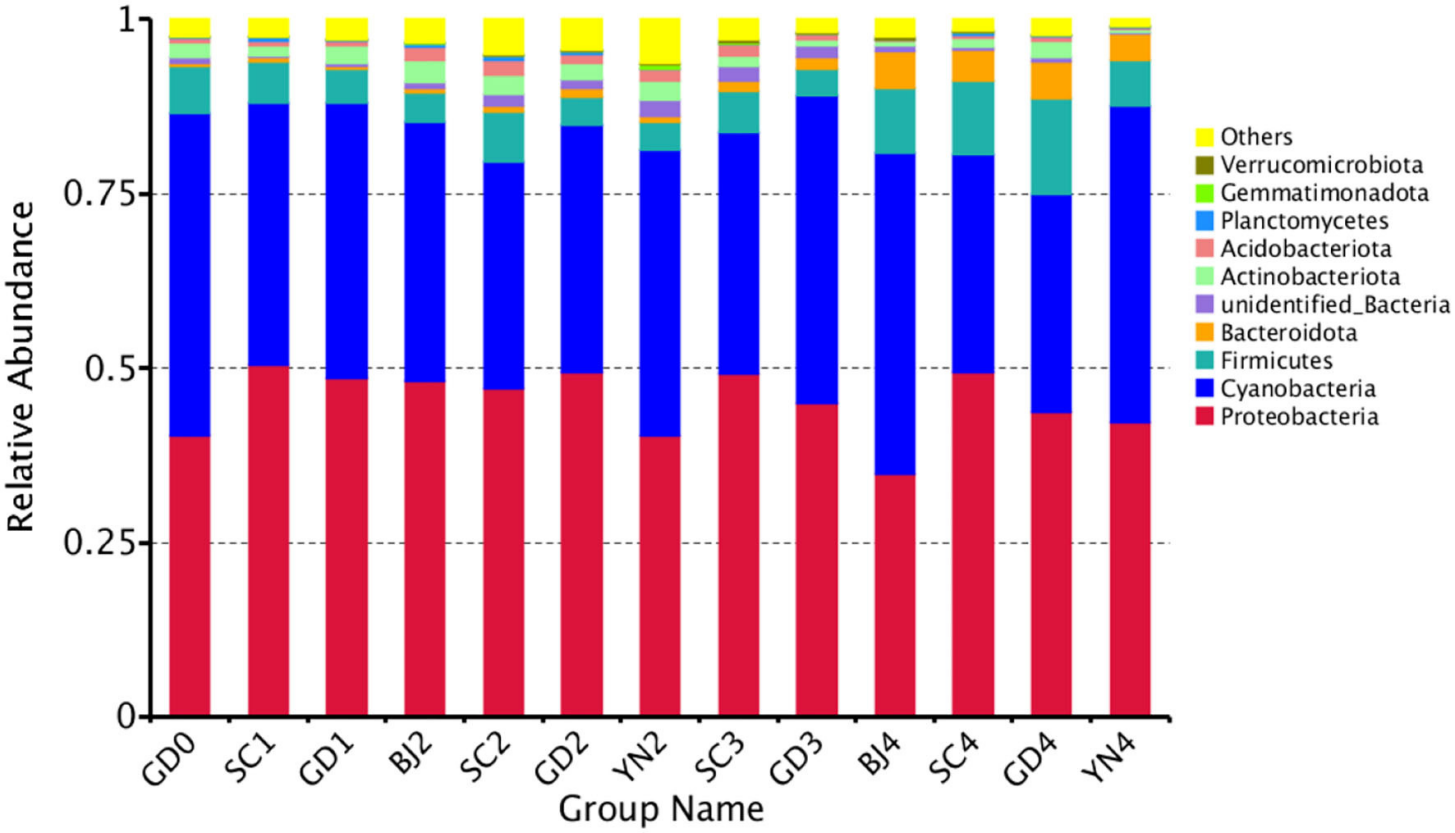
Relative abundance of bacterial at phylum levels. Distribution of 10 most abundant bacterial phylum present in *Citrus reticulata* “Chachi” samples. “Others” in the last column refers to the sum of less abundant bacterial phylum. Each number represents average of three replicates.

The composition of the bacterial communities identified on the PCR surface at each sampling point was analyzed and identified. Results are shown in Figure 7. It was found that the bacterial community composition on the surface of PCR was similar, but the abundance of the bacteria changed significantly during aging. At the phylum level, these bacteria were distributed mainly among the *Proteobacteria* (45.32%), *Cyanobacteria* (38.59%), *Firmicutes* (6.65%), *Bacteroidota* (2.1%), *Actinobacteriota* (1.89%), *Acidobacteriota* (0.99%), *Planctomycetes* (0.24%), *Verrucomicrobiota* (0.18%), and *Gemmatimonadota* (0.07%) (Figure 7).

**Figure 8:**
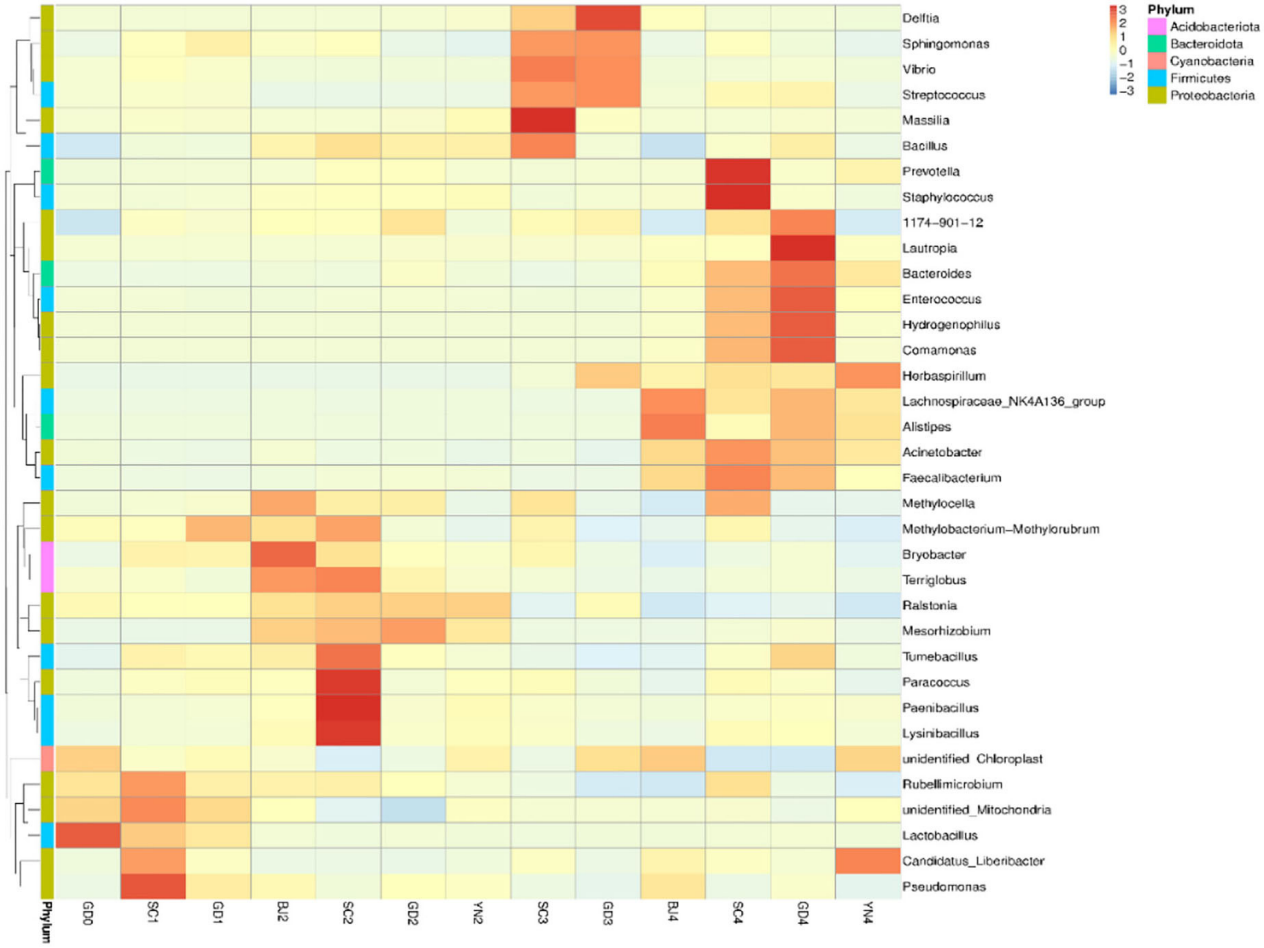
Heatmap depicting relative species abundance. Plotted by sample name on the X-axis and the Y-axis represents the genus. The cluster tree on the left side of the graph is a species cluster tree.

According to the results in Table 4 and Figure 8, all the known bacteria of the genera *Lactobacillus* were predominant in fresh citrus skin, while the genus *Pseudomonas* and *Lactobacillus* were abundantly distributed in SC1. *Methylobacterium* was predominant in GD1. *Bryobacter* and *Terriglobus* were predominant in BJ2. *Paenibacillus* and *Lysinibacillus* gathered more when samples were stored for SC2. *Mesorhizobium* and *Ralstonia* were predominant in GD2. *Ralstonia* was predominant in YN2. *Bacillus* and *Sphingomonas* were found in SC3, while *Delftia, Streptococcus, Vibrio*, and *Sphingomonas* gathered more when samples were stored for GD3. *Alistipes, Acinetobacter*, and *Pseudocercospora* were found in BJ4. *Staphylococcus* and *Prevotella* were predominant in SC4. *Lautropia* and *Hydrogenophilus* were predominant in GD4, while *Herbaspirillum* gathered more when samples were stored for YN4. Interestingly, *Methylobacterium, Sphingomonas, Bacillus*, and *Pseudomonas* accelerated the biotransformation of volatile oil in PCR. *Methylobacterium, Sphingomonas, Bacillus*, and *Pseudomonas* were dominant bacterial genera on the surface of PCR aged in Sichuan and Guangdong areas, indicating that Sichuan and Guangdong areas were suitable for natural aging of PCR.

**Table 4:** Distribution of the dominant bacterial genera in all samples.

**Sample name**	**The dominant bacterial genera**
GD0	*Lactobacillus*
SC1	*Pseudomonas, Rubellimicrobium, Candidatus, Liberibacter, Lactobacillus*
GD1	*Methylobacterium*
BJ2	*Bryobacter, Terriglobus, Methylocella, Mesorhizobium*
SC2	*Paenibacillus, Lysinibacillus, Paracoccus, Tumebacillus, Terriglobus, Methylobacterium, Mesorhizobium, Ralstonia*
GD2	*Mesorhizobium, Ralstonia*
YN2	*Ralstonia*
SC3	*Massilia, Vibrio, Bacillus, Sphingomonas, Streptococcus, Delftia*
GD3	*Delftia, Streptococcus, Vibrio, Sphingomonas, Herbaspirillum*
BJ4	*Alistipes, Acinetobacter, Faecalibacterium*
SC4	*Staphylococcus, Prevotella, Faecalibacterium, Acinetobacter, Methylocella, Comamonas, Hydrogenophilus, Enterococcus, Bacteroides, Herbaspirillum, Rubellimicrobium*
GD4	*Lautropia, Hydrogenophilus, Enterococcus, Comamonas, Alistipes, Faecalibacterium, Acinetobacter, Tumebacillus*
YN4	*Candidatus, Liberibacter, Herbaspirillum*

**Figure 9:**
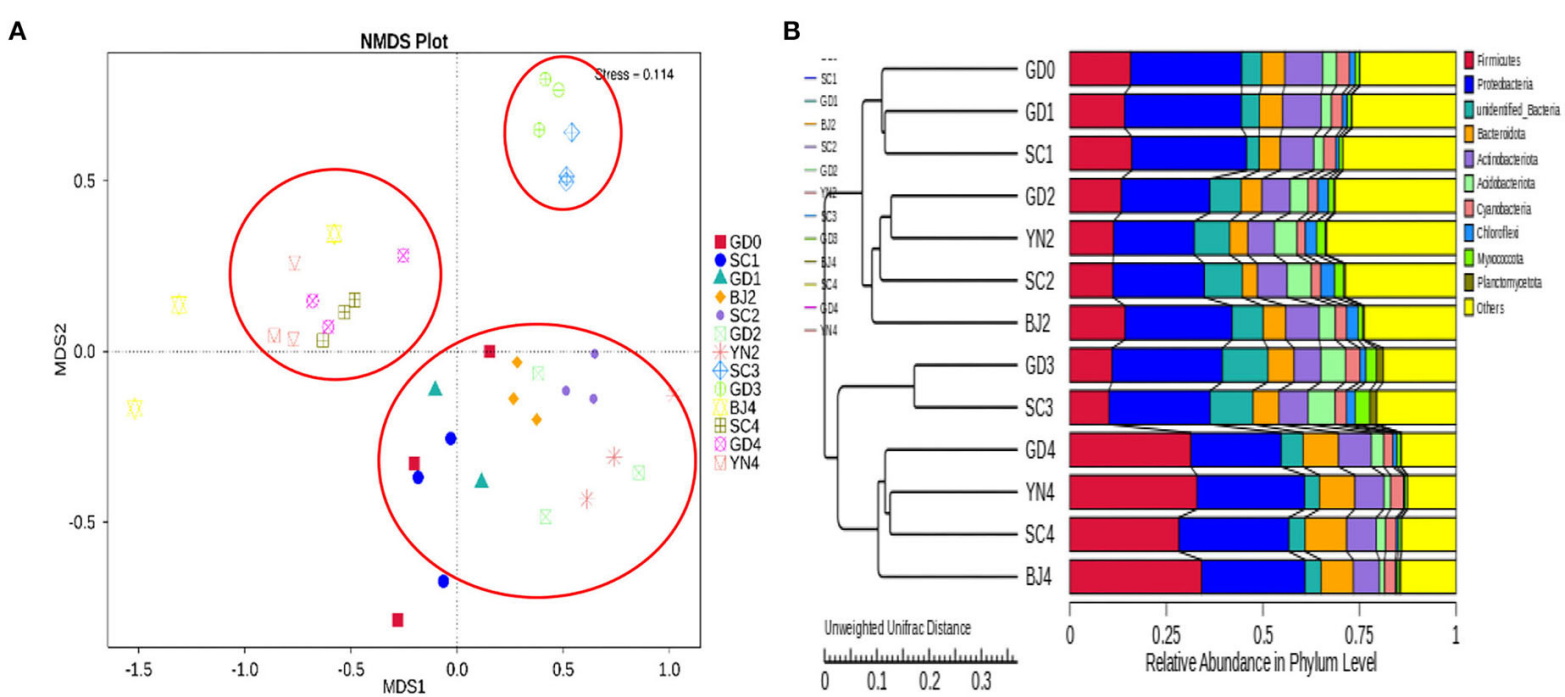
**(A)** NMDS analysis of fungal community composition. Each point represents a sample, plotted by the second principal component on the Y-axis and the first principal component on the X-axis, which was colored by group. **(B)** UPGMA clustering tree based on weighted UniFrac distance. UPGMA clustering tree (at left). The relative phylum-level abundance map (at right).

To evaluate the similarities and differences between these samples, we carried out an MNDS analysis. The obtained results are shown in Figure 9. It was found that the distances between the PCR samples with the same aging time were relatively small. According to the UPGMA clustering tree results, the composition of bacterial species was similar during the aging process and, as such, could be evaluated as a single group (Figure 9).

### GC/MS Analysis of Volatile Components

To compare volatile components of PCR samples at different stages, the chemical profiling of volatile components was analyzed by GC/MS. Steam distillation for volatile oil of PCR was prepared according to the method below 1.3, and GC/MS analysis was performed according to the method described in Section Materials and Methods. The total ion chromatogram (as shown in Supplementary Figure 1) and mass spectrum data of PCR volatile oil sample were obtained. The volatile compounds in PCR samples were identified by comparing their mass spectra with reference mass spectra from the National Institute of Standards and Technology (NIST) and related literature (Liu, 2018; Wang et al., 2018; Sheng et al., 2020). A total of 55 volatile compounds were identified, and there were no differences among the 55 volatile compounds of PCR samples at different stages. The relative percentage content of each component was calculated by the area normalization method. The CAS number, retention time, and relative contents of the identified volatile compounds are summarized in Table 5. The results indicated that D-Limonene (V9) was the most abundant volatile compound in PCR samples at different storage periods and aging stages with its relative content in the range of 65.51–72.64%, followed by gamma-Terpinene (V11) with a relative content of 14.32–16.81%. The total contents of these two volatile components accounted for about 90% of the volatile oil content.

**Table 5:** Volatile compounds of *Citrus reticulata* “Chachi” samples.

	**Compounds**	**CAS number**	**Retention time(min)**	**Relative amount (%)**				
				**GD0**	**SC1**	**GD1**	**BJ2**	**SC2**
V1	3-Thujene	002867-05-2	5.207	0.35	0.79	0.55	0.63	0.60
V2	alpha-Pinene	007785-70-8	5.403	1.34	2.32	1.70	2.13	2.00
V3	camphene	000079-92-5	5.85	0.02	0.02	0.02	0.02	0.02
V4	Sabenene	003387-41-5	6.667	0.41	0.33	0.27	0.38	0.37
V5	beta-Pinene	000127-91-3	6.768	1.31	1.54	1.30	1.59	1.54
V6	beta-Myrcene	000123-35-3	7.316	2.12	2.29	2.12	2.21	2.17
V7	alpha-Phellarene	000099-83-2	7.825	0.16	0.15	0.14	0.14	0.15
V8	a-Terpinene	000099-86-5	8.323	0.39	0.43	0.39	0.41	0.40
V9	D-Limonene	005989-27-5	9.083	65.51	72.19	72.48	70.95	70.61
V10	beta-Ocimene	013877-91-3	9.76	0.06	0.03	0.03	0.04	0.05
V11	gamma-Terpinene	000099-85-4	10.297	16.81	14.57	14.32	15.49	15.92
V12	Terpinolene	000586-62-9	11.618	1.21	0.81	0.83	0.90	0.95
V13	Bicyclo[3.1.0]hexan-2-ol,2-methyl-5-(1-methylethyl)-, (1R,2R,5S)-rel-(trans-4-Thujanol)	017699-16-0	12.289	0.03	0.03	0.03	0.04	0.02
V14	Linalool	000078-70-6	12.44	0.22	0.13	0.20	0.17	0.19
V15	1-Nonanal	000124-19-6	12.569	0.08	0.05	0.05	0.04	0.07
V16	2-Cyclohexen-1-ol,1-methyl-4-(1-methylethenyl)-, (1R,4R)-rel-(cis-p-Menth-2,8-diene-1-ol)	007212-40-0	13.424	0.05	0.03	0.03	0.03	0.02
V17	Citronellal	000106-23-0	14.42	0.08	0.05	0.07	0.06	0.06
V18	Terpinen-4-ol	000562-74-3	14.968	0.36	0.16	0.25	0.24	0.22
V19	alpha-Terpineol	000098-55-5	15.265	0.59	0.37	0.52	0.45	0.41
V20	Decanal	000112-31-2	15.489	0.37	0.19	0.22	0.22	0.32
V21	(Z)-Carveol	001197-06-4	15.768	0.06	0.02	0.03	0.02	0.02
V22	Citronellol	000106-22-9	15.897	0.06	0.03	0.04	0.04	0.03
V23	2-methoxy-4-methyl-1-(1-methylethyl)-Benzene	001076-56-8	15.947	0.06	0.04	0.04	0.04	0.04
V24	D(+)-Carvone	002244-16-8	16.104	0.05	0.02	0.03	0.02	0.03
V25	Piperitone	000089-81-6	16.255	0.02	-	-	-	-
V26	4-(1-methylethenyl)-1-Cyclohexene-1-carboxaldehyde	002111-75-3	16.54	0.19	0.09	0.12	0.11	0.11
V27	Thymol	000089-83-8	16.954	0.39	0.23	0.36	0.25	0.26
V28	5-Isopropyl-2-methylphenol	000499-75-2	17.094	0.19	0.11	0.18	0.23	0.14
V29	Citronellyl acetate	000150-84-5	17.642	0.03	-	-	-	-
V30	Neryl acetate	000141-12-8	17.838	0.04	0.01	0.01	0.01	0.01
V31	alpha.-Cubebene	017699-14-8	18.09	0.06	0.03	0.03	0.03	0.02
V32	Geranyl acetate	000105-87-3	18.168	0.02	-	-	0.01	0.01
V33	Cubebene	013744-15-5	18.347	0.08	0.05	0.05	0.05	0.04
V34	Dodecyl aldehyde	000112-54-9	18.627	0.14	0.06	0.06	0.06	0.09
V35	Methyl 2-(methylamino)benzoate	000085-91-6	18.789	2.20	1.12	1.63	1.40	1.40
V36	l-Caryophyllene	000087-44-5	18.957	0.24	0.13	0.14	0.15	0.16
V37	Humulene	006753-98-6	19.656	0.03	0.02	0.02	0.01	0.01
V38	Germacrene D	023986-74-5	20.193	0.03	0.02	0.02	0.02	0.01
V39	g-Selinene	000515-17-3	20.478	0.07	0.04	0.04	0.04	0.04
V40	a-Farnesene	000502-61-4	20.585	0.81	0.41	0.36	0.33	0.39
V41	Phenol,2,5-bis(1,1-dimethylethyl)-	005875-45-6	20.836	0.03	-	-	-	-
V42	d-Cadinene	000483-76-1	20.965	0.08	0.04	0.04	0.04	0.03
V43	a-Elemol	000639-99-6	21.502	0.03	0.02	0.02	0.01	0.01
V44	Dodecanoic acid	**000143-07-7**	21.726	0.04	-	0.01	-	-
V45	Caryophyllene oxide	**001139-30-6**	22.145	0.03	0.01	0.01	0.01	0.02
V46	Tetradecanal	000124-25-4	22.464	0.02	0.01	0.01	-	0.01
V47	a-Sinensal	017909-77-2	24.819	1.22	0.50	0.61	0.38	0.43
V48	Methyl hexadecanoate	000112-39-0	27.135	0.03	0.01	0.01	-	0.01
V49	Palmitic acid	000057-10-3	27.689	0.15	0.03	0.04	0.02	0.02
V50	Ethyl palmitate	000628-97-7	28.002	0.03	0.02	-	-	-
V51	Methyl linoleate	000112-63-0	29.278	0.02	-	0.01	-	-
V52	Methyl linolenate	000301-00-8	29.362	0.03	-	-	-	-
V53	ETHYL LINOLEATE	007619-08-1	30.055	0.10	0.03	-	0.01	0.02
V54	ethyl linolenate	001191-41-9	30.145	0.12	0.02	-	-	0.01
V55	2,2′-Methylenebis(6-tert-butyl-4-methylphenol)	000119-47-1	33.003	0.17	0.16	0.23	0.19	0.15
	化合物	GD2	YN2	SC3	GD3	BJ4	SC4	GD4	YN4
V1	3-Thujene	0.41	0.55	0.56	0.61	0.55	0.53	0.55	0.41
V2	alpha-Pinene	1.42	1.89	2.03	1.87	1.88	1.96	1.94	1.71
V3	camphene	0.02	0.02	0.02	0.02	0.02	0.02	0.02	0.02
V4	Sabenene	0.29	0.40	0.39	0.23	0.40	0.40	0.35	0.38
V5	beta-Pinene	1.31	1.50	1.56	1.40	1.42	1.43	1.44	1.45
V6	beta-Myrcene	2.02	2.11	2.26	2.33	2.20	2.27	2.28	2.04
V7	alpha-Phella-rene	0.11	0.15	0.13	0.11	0.12	0.11	0.11	0.11
V8	a-Terpinene	0.40	0.39	0.37	0.40	0.39	0.37	0.39	0.36
V9	D-Limonene	70.75	70.50	72.37	72.64	70.90	72.28	72.54	68.64
V10	beta-Ocimene	0.05	0.05	0.06	0.03	0.04	0.04	0.04	0.04
V11	gamma-Terpinene	16.55	15.67	15.49	15.25	15.16	15.74	15.25	16.21
V12	Terpinolene	1.00	0.93	0.94	0.93	0.89	0.94	0.88	1.01
V13	Bicyclo[3.1.0]hexan-2-ol,2-methyl-5-(1-methylethyl)-, (1R,2R,5S)-rel-(trans-4-Thujanol)	-	0.03	-	-	-	-	-	-
V14	Linalool	0.15	0.20	0.12	0.08	0.12	0.08	0.09	0.14
V15	1-Nonanal	0.04	0.06	0.04	0.03	0.03	0.03	0.03	0.04
V16	2-Cyclohexen-1-ol,1-methyl-4-(1-methylethenyl)-, (1R,4R)-rel-(cis-p-Menth-2,8-diene-1-ol)	0.02	0.03	0.01	0.02	0.02	0.01	0.01	0.02
V17	Citronellal	0.05	0.07	0.06	0.03	0.06	0.04	0.03	0.06
V18	Terpinen-4-ol	0.21	0.26	0.15	0.16	0.22	0.11	0.15	0.22
V19	alpha-Terpineol	0.34	0.46	0.24	0.22	0.37	0.16	0.22	0.33
V20	Decanal	0.23	0.31	0.22	0.16	0.17	0.19	0.16	0.28
V21	(Z)-Carveol	0.02	0.02	0.02	0.01	0.02	0.01	0.01	0.01
V22	Citronellol	0.03	0.04	0.02	0.02	0.02	-	0.01	0.02
V23	2-methoxy-4-methyl-1-(1-methylethyl)-Benzene	0.04	0.05	0.03	0.03	0.04	0.02	0.03	0.04
V24	D(+)-Carvone	0.02	0.03	0.02	0.01	0.02	0.01	0.01	0.02
V25	Piperitone	-	0.01	-	-	-	-	-	-
V26	4-(1-methylethenyl)-1-Cyclohexene-1-carboxaldehyde	0.09	0.11	0.07	0.06	0.08	0.05	0.06	0.11
V27	Thymol	0.29	0.30	0.15	0.14	0.26	0.15	0.14	0.29
V28	5-Isopropyl-2-methylphenol	0.10	0.15	0.08	0.05	0.23	0.08	0.06	0.17
V29	Citronellyl acetate	0.01	0.01	0.01	-	0.01	0.01	-	0.01
V30	Neryl acetate	0.02	0.02	0.01	0.01	0.02	0.01	0.01	0.02
V31	alpha.-Cubebene	0.03	0.03	0.03	0.03	0.04	0.05	0.04	0.05
V32	Geranyl acetate	0.01	0.01	-	-	-	-	-	0.01
V33	Cubebene	0.04	0.05	0.04	0.05	0.05	0.05	0.05	0.08
V34	Dodecyl aldehyde	0.08	0.08	0.07	0.06	0.06	0.07	0.06	0.11
V35	Methyl 2-(methylamino)benzoate	1.62	1.45	1.06	0.94	1.48	0.79	0.91	1.69
V36	l-Caryophyllene	0.20	0.16	0.13	0.15	0.21	0.18	0.17	0.23
V37	Humulene	0.02	0.02	0.02	0.01	0.02	0.02	0.02	0.03
V38	Germacrene D	0.02	0.02	0.02	0.02	0.02	0.02	0.02	0.03
V39	g-Selinene	0.05	0.05	0.05	0.05	0.05	0.06	0.05	0.07
V40	a-Farnesene	0.50	0.46	0.35	0.43	0.58	0.56	0.48	0.75
V41	Phenol,2,5-bis(1,1-dimethylethyl)-	-	-	-	-	-	-	-	-
V42	d-Cadinene	0.05	0.04	0.05	0.05	0.06	0.06	0.06	0.08
V43	a-Elemol	0.02	0.02	0.01	0.01	0.01	0.01	0.01	0.03
V44	Dodecanoic acid	0.01	-	0.01	-	-	0.01	-	0.03
V45	Caryophyllene oxide	0.02	0.02	0.02	0.02	0.02	0.01	0.02	0.02
V46	Tetradecanal	0.01	0.01	0.01	0.01	-	0.01	0.01	0.01
V47	a-Sinensal	0.67	0.53	0.29	0.65	0.77	0.54	0.63	1.13
V48	Methyl hexadecanoate	0.01	0.01	-	-	-	0.01	-	0.01
V49	Palmitic acid	0.03	0.03	0.03	0.08	0.02	0.04	0.08	0.15
V50	Ethyl palmitate	0.01	0.01	0.01	-	-	0.01	0.01	0.04
V51	Methyl linoleate	-	-	0.01	-	0.01	0.01	0.01	0.02
V52	Methyl linolenate	-	-	-	-	-	0.01	-	-
V53	ETHYL LINOLEATE	0.04	0.03	0.02	0.02	0.02	0.03	0.03	0.10
V54	ethyl linolenate	0.03	0.02	0.02	0.02	0.01	0.03	0.02	0.07
V55	2,2′-Methylenebis(6-tert-butyl-4-methylphenol)	0.16	0.18	0.13	0.19	0.15	0.13	0.21	0.27

*Compounds identified via GC/MS analysis based on the comparison with retention indices (RI) and the mass spectra of the standard compounds (similarity ≥ 85%). Relative amount, the percentage of each compound area to total area of identified compounds; “-”, Not detected. Results are presented as means (n = 3)*.

It is clear that the volatile compounds present in the volatile oils of PCR samples at different stages were the same, but their relative contents were different. The results indicated that the variation in the volatile chemical compositions occurred during aging. To further compare the difference in volatile components of PCR samples at different developmental stages, multivariate analysis methods, including PCA and OPLS-DA, were performed based on the relative contents of the 55 volatile compounds. This allowed us to find out the different components in the volatile oil samples of PCR at different stages, and to clarify the dynamic change process of volatile components of PCR.

#### Principal Component Analysis

**Figure 10:**
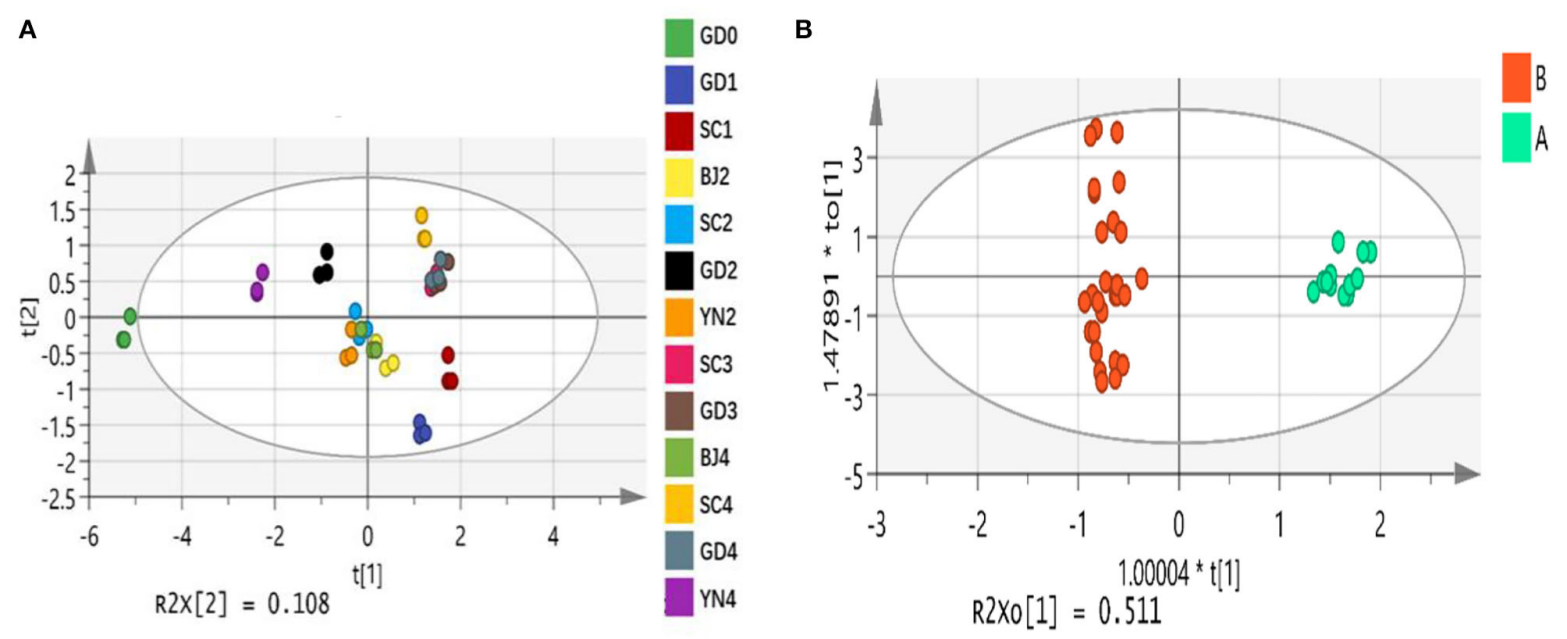
**(A)** The principal component analysis scores plot of *Citrus reticulata* “Chachi” volatile oil samples in different aging time. **(B)** The orthogonal partial least square-discriminate analysis scores plot of *Citrus reticulata* “Chachi” volatile oil samples in different aging time.

PCA is an unsupervised pattern recognition method. PCA could be used for analyzing, classifying, and reducing the dimensionality of numerical datasets, and has been widely used in discriminating and comparing the composition of samples from different areas, different varieties, and different harvest times (Huang et al., 2018b; Chen et al., 2019; Xue et al., 2019). PCA was first employed to visualize the grouping trends based on the relative contents of the 55 volatile components. The first and second principal components described 70.3% and 10.8% of the variability in the original observations, and the first two principal components accounted for 81.1% of the total variance. Our analysis showed that the established PCA model had a good distinction. The first two principal components reflected the main characteristics of the PCR samples. As shown in Figure 10A, SC3, GD3, SC4, and GD4 were distant from those represented in the other samples.

#### Orthogonal Partial Least Squares Discriminant Analysis

OPLS-DA is a supervised pattern recognition method. To find potential differential chemical markers that contributed to the differences among PCR samples, supervised OPLS-DA was used based on the relative contents of the 55 volatile components. The explanatory ability parameters of OPLS-DA were 0.957. The predictive ability parameter was 0.989, which revealed a good classification and prediction ability of the model. Similar to the PCA results, the OPLS-DA scores plot (Figure 10B) showed that the PCR samples could also be readily classified into six groups according to six different developmental stages. To select the differential chemical markers, the 55 volatile compounds were further screened based on variable importance in projection (VIP) values. The VIP values represented the differences between the variables, and components that played important roles in differentiation were picked out when the VIP values were more than 1.0. A total of ten volatile compounds, including alpha-Pinene (VIP = 1.33), beta-Myrcene (VIP = 1.23), D-Limonene (VIP = 4.79), gamma-Terpinene (VIP = 2.04), Terpinen-4-ol (VIP = 1.03), alpha-Terpineol (VIP = 1.61), Thymol (VIP=1.32), 5-Isopropyl-2-methylphenol (VIP = 1.01), Methyl 2-(methylamino)benzoate (VIP = 2.72) and a-Sinensal (VIP = 1.41)with their VIP values (>1.0) were selected as chemical markers that were responsible for the significant intergroup differences of PCR samples.

Further, we analyzed and compared the dynamic change process of volatile components of 10 chemical markers. The relative contents of gamma-terpinene, Terpinen-4-OL, alpha-Terpineol, Thymol, Methyl 2-(methylamino) Benzoate and a-Sinensal reached the highest peaks in fresh peels. The relative contents of alpha-Pinene reached the highest values in GD1. The relative content of 5-isopropyl-2-methylphenol changed little while the relative content of beta-Myrcene and D-Limonene reached the highest in GD3.

### Spearman-Based Correlation Analysis Between Microbiota and Chemical Markers

**Figure 11:**
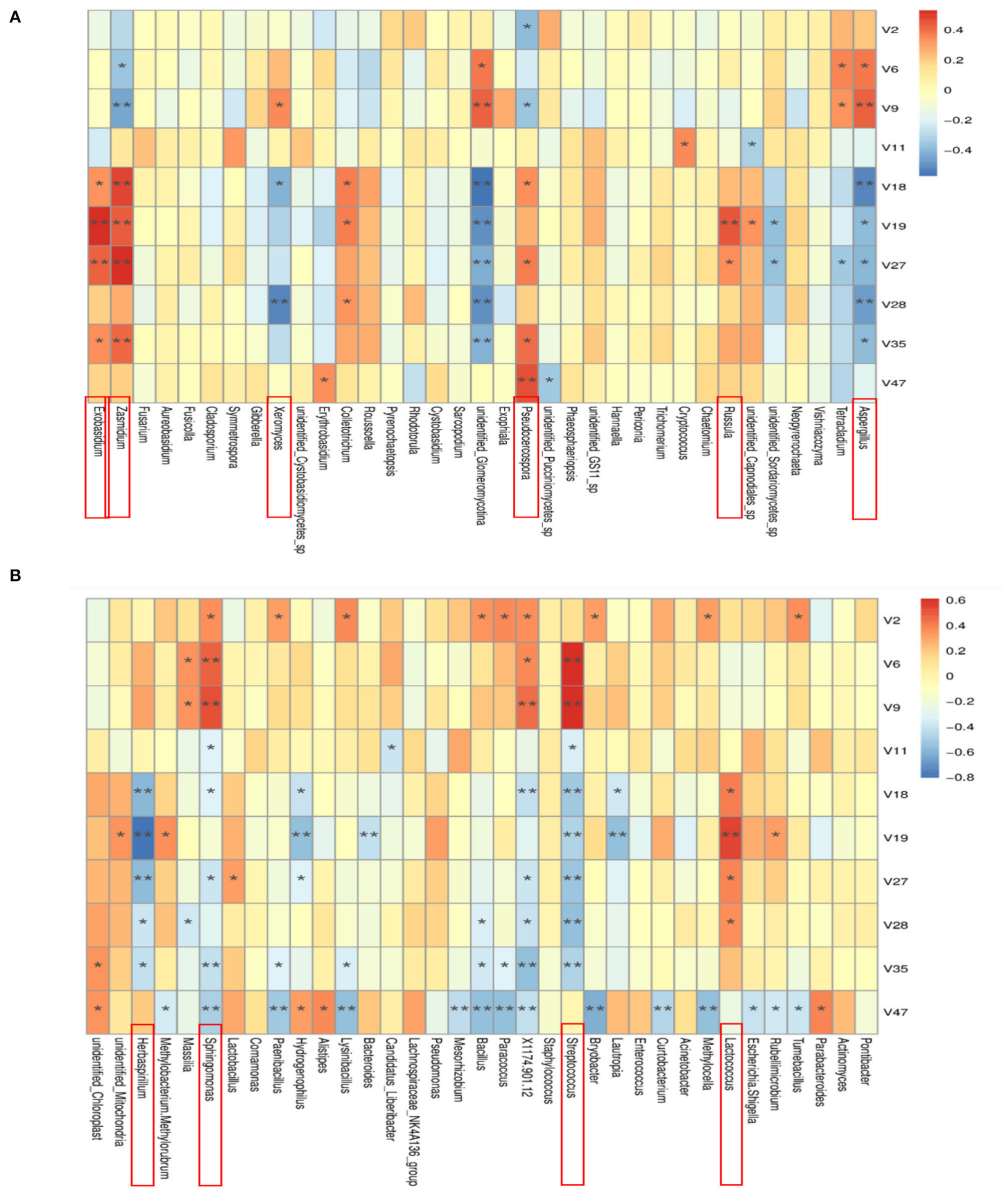
Spearman correlation analysis heat map between the top 35 bacteria **(A)** and fungi **(B)** in relative abundance and the chemical markers that change significantly during aging of *Citrus reticulata* “Chachi”. The closer r is to 1 (red), it indicates a positive correlation, and the closer to −1 is a negative correlation (blue). The asterisk represented the level of significance, “*” represented *p* < 0.05, “**” represented *p* < 0.01.

Spearman analysis was used to analyze the correlation between the relative abundance of the top 35 microbial genera and the PCR chemical markers. Results are shown in Figure 11. All known fungi of the genera, *Exobasidium, Zasmidium, Xeromyces, Pseudocercospora, Russula*, and *Aspergillus* were strongly correlated with chemical markers. Among them, *Aspergillus* was strongly correlated with most of the selected chemical markers. In detail, it was positively correlated with beta-Myrcene and D-Limonene and negatively correlated with Terpinen-4-ol, alpha-Terpineol, Thymol, 5-Isopropyl-2-methylphenol, and Methyl 2-(methylamino)benzoate. *Aspergillus*, especially *Aspergillus niger*, played an important role in the change of flavonoids and volatile oil in PCR. Data show that bio-augmented fermentation with *A. niger* NCUF413 was proven to enhance the liquor yield and improve the flavor of the liquor samples (Pan et al., 2020). All the known bacteria of the genera, *Herbaspirillum, Sphingomonas, Streptococcus*, and *Lactococcus* were strongly correlated with chemical markers. Among them, *Sphingomonas* was positively correlated with alpha-Pinene, beta-Myrcene, and D-Limonene and negatively correlated with gamma-Terpinene, Terpinen-4-ol, Thymol, Methyl 2-(methylamino)benzoate and a-Sinensal. *Lactococcus* was positively correlated with Terpinen-4-ol, alpha-Terpineol, Thymol, and 5-Isopropyl-2-methylphenol. *Sphingomonas*, as the dominant microbial genera of tobacco, was also proven to increase tobacco aroma. In our case, *Sphingomonas* and *Lactococcus* accelerated the biotransformation of volatile oil in PCR.

## Conclusions

Aging of PCR is very important to improve its quality. However, the natural aging process of PCR takes a long time and costs a lot. At present, the use of microbial transformation to increase active components and improve quality is widely studied. Therefore, after clarifying the correlation between volatile contents and microbial population of PCR, we can explore whether there is a method of “the artificial aging technology” to shorten the aging time, reduce economic loss, and waste of resources will be useful. In addition, microbial diversity is abundant, and their growth, physiological and biochemical characteristics will be unstable with the change in the environment. Pericarpium Citri Reticulatae can provide nitrogen and carbon sources for microbial respiration, PH alteration caused by the acid-producing microbes also affects microbial growth. Further exploring the microbial succession and its relative environmental factors could provide valuable information for understanding the mechanism of Pericarpium Citri Reticulatae aging process.

In this study, the microbial population structure and volatile components of the 1-year aging process of PCR from Beijing, Sichuan, Guangdong, and Yunnan were analyzed. The microbial population structure in *Citrus reticulata* “Chachi” with different aging times was consistent in composition and structure but the dominant microorganisms on its surface were different significantly. GC/MS was applied to the analysis of volatile components, and 55 volatile compounds were detected. SC3, GD3, SC4, and GD4 were distant from those representing the other samples, alpha-pinene, beta-myrcene, D-Limonene, and other 10 chemical markers were significantly different from other samples. This indicated that volatile components' relative contents were similar in areas with similar environmental factors. We further proved that *Exobasidium, Xeromyces, Pseudocercospora, Russula, Aspergillus, Herbaspirillum, Sphingomonas*, and *Streptococcus* were the dominant microbial genera in Sichuan and Guangdong, and their abundance further showed strong connections with volatile components of chemical markers. Our results allow us to highlight the key influencing factors of its aging quality. To further clarify their exact contribution, using pure strains of these microorganisms to perform fermentation, is required to validate their applications.

## Data Availability Statement

The authors acknowledge that the data presented in this study must be deposited and made publicly available in an acceptable repository, prior to publication. Frontiers cannot accept a article that does not adhere to our open data policies.

## Author Contributions

FY wrote the manuscript. LH and MS analyzed data. FW modified the details. HC and YL conceived of or designed the study. All authors agreed on the contents of the paper.

## Funding

This research was supported by grants from the National Natural Science Foundation of China (No.81973436).

## Conflict of Interest

The authors declare that the research was conducted in the absence of any commercial or financial relationships that could be construed as a potential conflict of interest.

## Publisher's Note

All claims expressed in this article are solely those of the authors and do not necessarily represent those of their affiliated organizations, or those of the publisher, the editors and the reviewers. Any product that may be evaluated in this article, or claim that may be made by its manufacturer, is not guaranteed or endorsed by the publisher.

## Supplementary Material

Supplementary Figure 1 The typical total ion chromatogram of PCR volatile oil sample by GC/MS.
