# Supplementary figures and images for: A Correlation Between Pericarpium Citri Reticulatae Volatile Components and the Change of the Coexisting Microbial Population Structure Caused by Environmental Factors During Aging
Source: Front Microbiol. 2022 Jul 22;13:930845. doi: 10.3389/fmicb.2022.930845 (PMC9355980; doi:10.3389/fmicb.2022.930845)

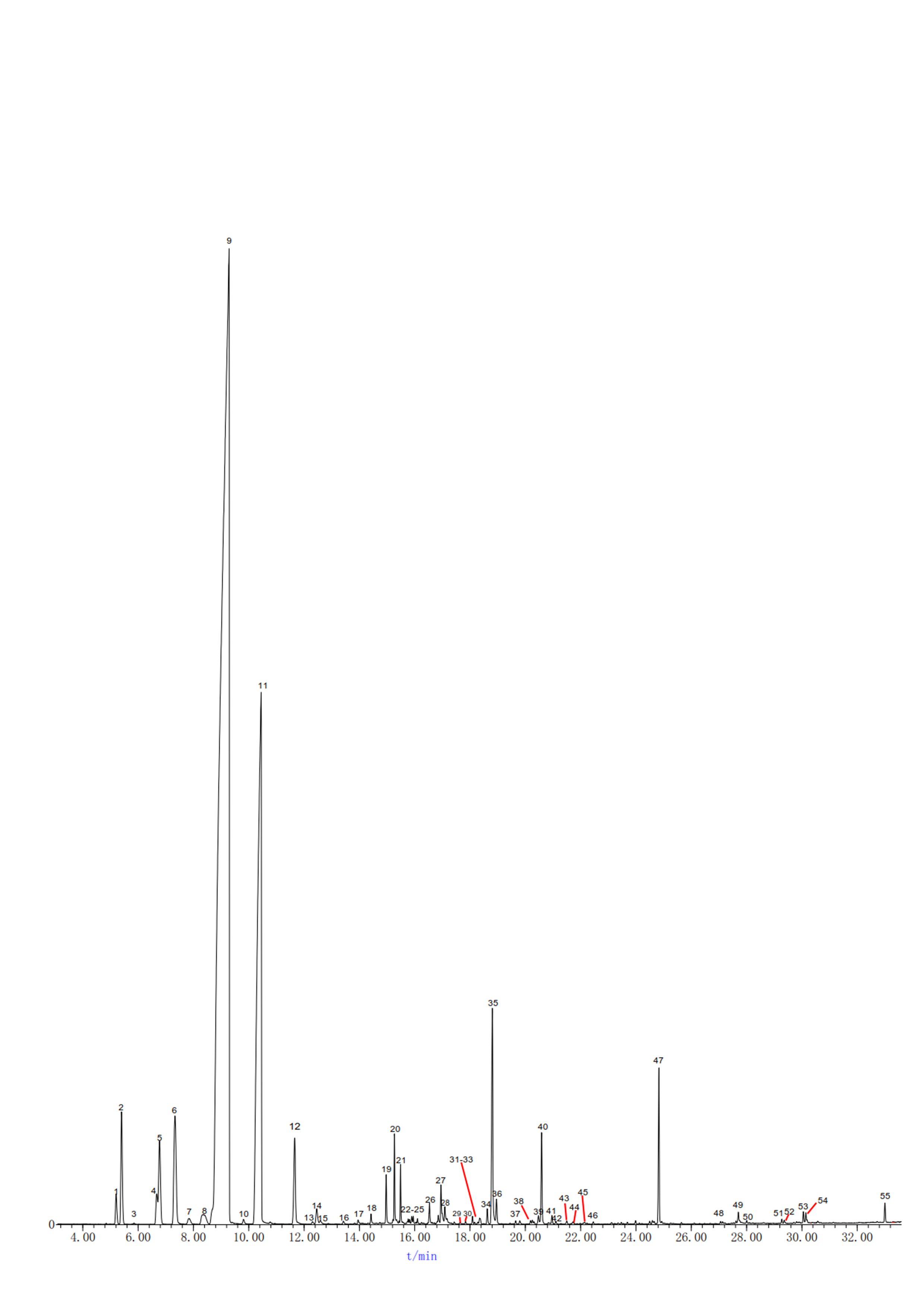

Supplement: Supplementary Figure 1 — The typical total ion chromatogram of PCR volatile oil sample by GC/MS. [file Image_1.TIF]
